# Supplementary material for: Immune-Mediated Bidirectional Causality Between Inflammatory Bowel Disease and Chronic Periodontitis: Evidence from Mendelian Randomization and Integrative Bioinformatics Analysis
Source: Biomedicines. 2025 Feb 15;13(2):476. doi: 10.3390/biomedicines13020476 (PMC11853167; doi:10.3390/biomedicines13020476)
Supplement: Supplementary file 1 [file biomedicines-13-00476-s001.zip › Figure S1. Funnel plot for the study.pdf]

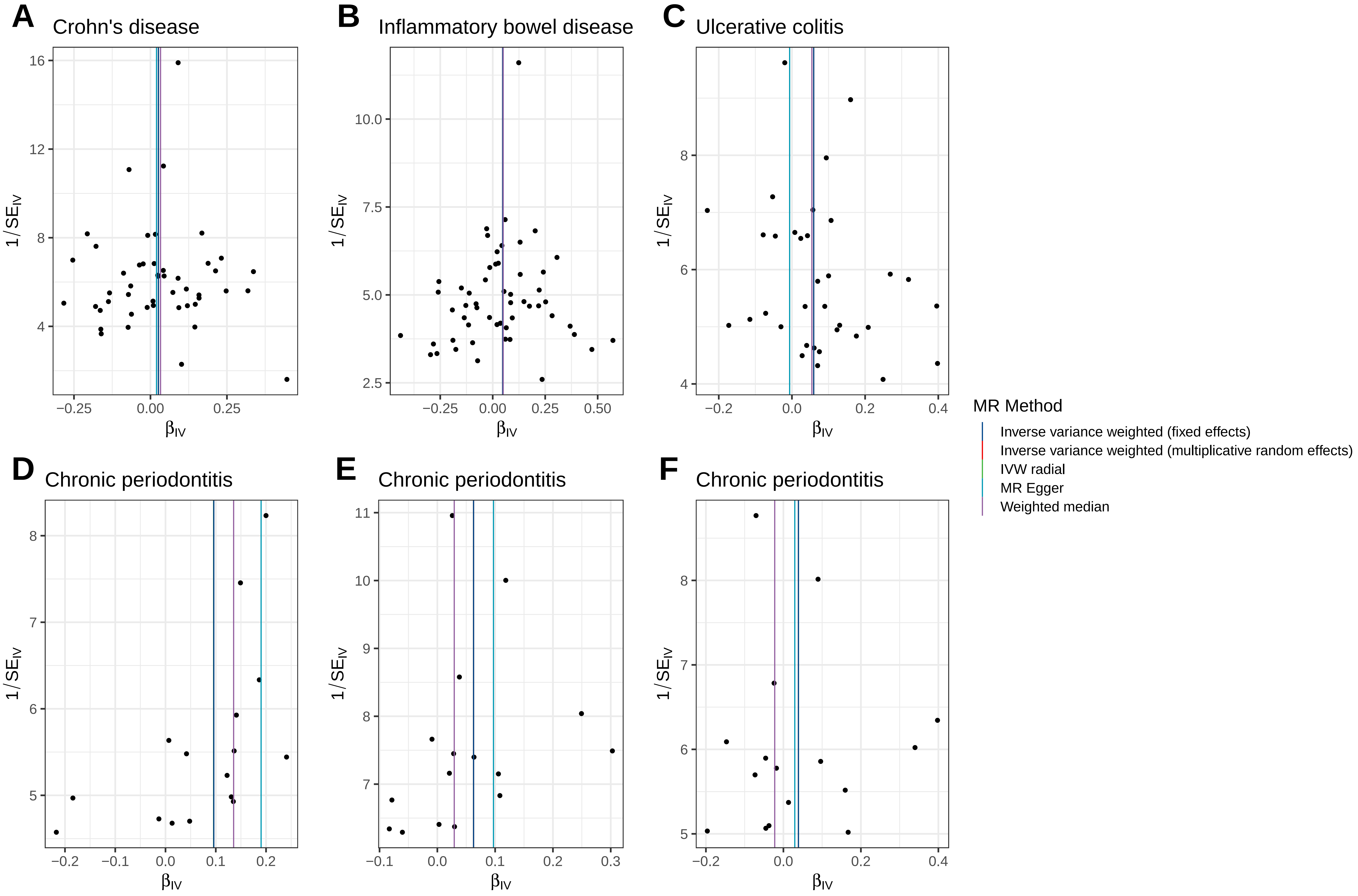

A
Crohn's disease
B
Inflammatory bowel disease
C
Ulcerative colitis
D
Chronic periodontitis
E
Chronic periodontitis
F
Chronic periodontitis
1/SE_IV
β_IV
MR Method
Inverse variance weighted (fixed effects)
Inverse variance weighted (multiplicative random effects)
IVW radial
MR Egger
Weighted median
